# Fecal Microbiota Characteristics in Constipation-Predominant and Mixed-Type Irritable Bowel Syndrome

**DOI:** 10.3390/microorganisms12071414

**Published:** 2024-07-12

**Authors:** Mariya Gryaznova, Yuliya Smirnova, Inna Burakova, Polina Morozova, Svetlana Lagutina, Pavel Chizhkov, Olga Korneeva, Mikhail Syromyatnikov

**Affiliations:** 1 Laboratory of Metagenomics and Food Biotechnology, Voronezh State University of Engineering Technologies, 394036 Voronezh, Russia; mariya-vg@mail.ru (M.G.); dyd16@mail.ru (Y.S.); vitkalovai@inbox.ru (I.B.); ms.cloud00.00@mail.ru (P.M.); korneeva-olgas@yandex.ru (O.K.); 2 Department of Genetics, Cytology and Bioengineering, Voronezh State University, 394018 Voronezh, Russia; qooleer@yandex.ru; 3 Department of Polyclinic Therapy, Voronezh State Medical University Named after N.N. Burdenko, 394036 Voronezh, Russia; svlagutina97@mail.ru

**Keywords:** IBS subtypes, intestinal disorders, microbiome, next-generation sequencing, *16S rRNA*

## Abstract

Background: Irritable bowel syndrome (IBS) is a common condition that affects the lifestyle of patients. It is associated with significant changes in the composition of the gut microbiome, but the underlying microbial mechanisms remain to be fully understood. We study the fecal microbiome of patients with constipation-predominant IBS (IBS-C) and mixed-type IBS (IBS-M). Methods: We sequenced the V3 region of the *16S rRNA* on the Ion Torrent PGM sequencing platform to study the microbiome. Results: In the patients with IBS-C and IBS-M, an increase in alpha diversity was found, compared to the healthy group, and differences in beta diversity were also noted. At the phylum level, both IBS subtypes showed an increase in the Firmicutes/Bacteroidetes ratio, as well as an increase in the abundance of Actinobacteria and Verrucomicrobiota. Changes in some types of bacteria were characteristic of only one of the IBS subtypes, while no statistically significant differences in the composition of the microbiome were detected between IBS-C and IBS-M. Conclusions: This study was the first to demonstrate the association of *Turicibacter sanguinis*, *Mitsuokella jalaludinii*, *Erysipelotrichaceae UCG-003*, *Senegalimassilia anaerobia*, *Corynebacterium jeikeium*, *Bacteroides faecichinchillae*, *Leuconostoc carnosum*, and *Parabacteroides merdae* with IBS subtypes.

## 1. Introduction

Irritable bowel syndrome (IBS) is a functional gastrointestinal disorder characterized by abdominal pain and changes in bowel habits [1]. IBS is associated with a high burden of disease, accounting for at least 20% of all visits to outpatient gastroenterology clinics [2]. IBS reduces the quality of life, productivity at work, and also productivity at school [3]. According to the Rome IV criteria, a diagnosis of IBS is based on the presence of the following key features: recurrent abdominal pain; symptoms must have started at least six months before diagnosis; and they must be associated with two or more of the following symptoms: pain associated with bowel movement, change in fecal frequency, change in appearance or shape of feces [4]. Based on the Bristol Stool Form Scale (BSFS), IBS can be divided into subclasses [5,6]. The subclasses of IBS include constipation-predominant IBS (IBS-C), diarrhea-predominant IBS (IBS-D), mixed-type IBS (IBS-M), and unclassified IBS (IBS-U). Regardless of the subclassification, the condition is typically associated with higher levels of distress, reduced quality of life and work productivity, and increased healthcare costs and use of medical resources [2,7,8].

After a long study of the functions and roles of microorganisms in various life processes of the host organism, it has become clear that changes in the microbial composition in the human gut can cause the development of various intestinal pathologies, including IBS. Several independent, consistent studies indicate that the gut microbiome in IBS is depleted of *Bifidobacterium*, *Lactobacillus*, *Faecalibacterium*, and *Clostridium* compared to healthy controls [9,10,11]. While *Veillonella*, *Ruminococcus*, and pro-inflammatory species such as *Enterobacteriaceae* increase [12,13,14,15,16].

Several studies have reported that *Collinsella aerofaciens* is overrepresented in the gut microbiota of IBS patients, especially in IBS-D [17]. *C. aerofaciens* produces hydrogen (H2), ethanol, and formate as the main end products of glucose metabolism. Hydrogen is one of the most abundant gases produced by bacteria in the human colon and has been shown to reduce colonic transit [18]. Accordingly, hydrogen production in the gut is associated with IBS [19,20].

Representatives of the *Dialister* genus are also, according to the literature, associated with IBS in adult patients [21,22]. This is confirmed by a study conducted using metagenomic analysis (*16S rRNA*), where an increase in the number of bacteria of the genus *Dialister* was noted in patients with IBS [23]. In addition, some studies show a relationship between an increased *Dialister* content and the severity of the disease [24]. However, there was a negative correlation between the *Dialister* bacterium and the clinical symptoms of patients with the IBS-D subtype [25].

There is also the genus *Dorea*, in which a by-product of vital activity is a gaseous substance that causes abdominal pain and flatulence. The increase in members of this genus is closely linked to increased intestinal permeability, which is one of the physiological manifestations of the disease [26,27]. However, the available data are often contradictory; a recent systematic review of the gut microbiota in IBS patients showed an increase in the abundance of members of the *Lactobacillaceae* family and the *Bacteroides* genus [28]. Higher and lower Firmicutes/Bacteroidetes ratios have been reported in patients with IBS. These ratios are a rough measure of an altered microbial population [29,30]. Reduced gut microbiome diversity and the presence of *Clostridiales*, *Prevotella*, and methanogenic species have been proposed as signatures of an IBS-specific microbiome associated with symptom severity [31]. There is currently no standard treatment for IBS, and patients with different subtypes of the disease may require different treatments. In addition, there are still knowledge gaps and controversies regarding changes in the gut microbiota composition in IBS.

Therefore, there is a clear need to study the changes in the microbiome in IBS in detail to identify markers of this disease, which, in the future, will reduce the healthcare burden and improve the quality of life of patients through the development of effective therapies. We hypothesized that a comparison of the intestinal metagenomics features of healthy people and patients with IBS-C and IBS-M would allow us to more specifically characterize the differences between these groups and possibly help in the search for new microbiota markers associated with the development of IBS. Thus, this study aimed to investigate the fecal microbiome of patients with IBS-C and IBS-M using next-generation sequencing.

## 2. Materials and Methods

### 2.1. Ethical Principles

All procedures for studies involving patients followed the ethical standards of the committee responsible for human experimentation (institutional and national) and the 1964 Declaration of Helsinki. Informed consent to participate in this study was obtained from all patients. The experiment was conducted in accordance with the recommendations of the Ethics Committee for Biomedical Research of Voronezh State University (Protocol No. 42-02, dated 10 April 2023).

### 2.2. Objects of Study

The control group consisted of 12 male patients without gastrointestinal pathology. A total of 32 patients with a confirmed diagnosis of IBS were divided into 2 study groups according to the subtype of the disease: there were 14 patients in the IBS-C group and 18 patients in the IBS-M group (Table 1). This sample size is justified by the fact that some previous studies on the characteristics of the fecal microbiota in IBS patients were conducted with relatively small sample sizes [32,33,34].

All participants gave written consent for their anonymized personal data to be used for research purposes. Fecal samples were used to study the microbiome.

The diagnosis of IBS was made based on a series of complaints, anamnesis, and clinical symptoms (classified according to the Rome IV criteria 2016), as well as laboratory diagnostic data. The treatment of IBS patients was based on the use of the following groups of drugs: proton pump inhibitors (*Esomeprazole magnesium trihydrate*/*omeprazole*/*Pantoprazole sodium sesquihydrate*/*Rabeprazole sodium*), laxatives (*polyethylene glycol*/*Bisacodyl*/*Lactulose*/*Prucalopride*), nootropics (*Aminophenylbutyric* acid) and antispasmodics (*Drotaverine hydrochloride*/*Mebeverine hydrochloride*/*Papaverine hydrochloride*), and antidepressants (*Sertraline hydrochloride*).

Exclusion criteria for all patients were the following: use of antibiotics or pro- or prebiotics in the six months prior to this study; surgery on the gastrointestinal tract in the six months before this study; chronic diseases such as inflammatory bowel disease (IBD), celiac disease, metabolic or endocrine disorders; severe mental disorders; pregnancy and lactation in women; specific dietary restrictions (e.g., vegan, ketogenic diet); drug use; recent or ongoing infectious bowel disease such as gastroenteritis; alcohol abuse; uncontrolled diabetes.

Fecal samples (approximately 1 g) were collected after defecation in an Eppendorf tube using disinfected plastic equipment. The samples were immediately refrigerated and transported to the laboratory under temperature control for further testing.

### 2.3. Ion Torrent PGM Sequencing

Total DNA was extracted from each fecal sample using a commercially available HiPure Microbiome DNA Kit according to the protocol (Magen, Guangzhou, China). The amount of total DNA obtained from the samples was determined using a Qubit 2.0 Fluorometer (ThermoFisher, Waltham, MA, USA) and a Qubit dsDNA HS Assay Kit (Invitrogen, Waltham, MA, USA). Quality was assessed by agarose gel electrophoresis. We chose the V3 region of the *16S rRNA* gene as a target DNA segment for microbiome research. The selected fragment was amplified by real-time PCR using universal primers 337F (5′-GACTCCTACGGGAGGCWGCAG-3′) and 518R (5′-GTATTACCGCGGCTGCTGG-3′). PCR amplification was performed using 5X ScreenMix-HS (Evrogen, Moscow, Russia) with the following protocol: denaturation at 94 °C—4 min, then 37 cycles; 94 °C—30 s; 59 °C—30 s; 72 °C—30 s; with a final elongation at 72 °C—5 min. The products obtained after PCR amplification were purified using AMPure XP magnetic particles (Beckman Coulter, Brea, CA, USA) and used to prepare sequencing libraries.

Library preparation for sequencing on the Ion Torrent PGM platform (ThermoFisher, Waltham, MA, USA) was performed according to the manufacturer’s protocol for the NEBNext Fast DNA Library Prep kit (New England Biolabs, Ipswich, MA, USA). In the first step of library preparation, end-processing was performed, then adapters were applied, and the final libraries were purified using AMPure XP (Beckman Coulter, Brea, CA, USA). After obtaining purified sequencing libraries for the Ion Torrent PGM platform, their concentrations were measured by quantitative PCR using the QIAgen library concentration kit (QIAgen, Hilden, Germany).

The resulting libraries were mixed in equimolar quantities for emulsion PCR using the Ion PGM Template OT2 200 Kit on a OneTouch 2 instrument (Thermo Fisher Scientific, Waltham, MA, USA). The enrichment rate of the beads was 24%, which is in the optimal range of 10–30%.

Libraries were sequenced on the Ion Torrent PGM platform using the commercial Ion PGM Hi-Q View Sequencing Kit (Thermo Fisher Scientific, Waltham, MA, USA).

### 2.4. Bioinformatics and Statistical Analysis

Reads for each sample were obtained in BAM format and converted to FASTQ format (FileExporter plugin). Raw sequencing data were available from the NCBI BioProject database (BioProjectID: PRJNA 817720). Bioinformatics processing and phylogenetic analysis of the obtained data were performed using the R programming language in the R-studio environment (VSEARCH v.2.8.2 program). We used DADA2 1.32.0 version for quality filtering of the raw reads. This step involved removing low-quality reads, trimming reads based on quality scores, and filtering out reads with ambiguous bases. Filtering was performed using a maximum expected error threshold of 1.0 [35]. We then performed dereplication by combining all identical sequencing reads into unique sequences. Although DADA2 provides amplicon sequence variants (ASVs) directly, we also performed OTU clustering using phyloseq to group similar sequences, reducing the impact of sequencing errors [36]. We assigned taxonomy to the sequence variants using the assignTaxonomy function from the DADA2 package, which provides a comprehensive and accurate taxonomic classification. Species-level taxonomy was determined with 97% identity to amplicon sequence variants using version 138.1 of the SILVA database (https://www.arb-silva.de, accessed on 29 April 2024). The resulting phylogenetic data were combined using the phyloseq package 1.38.0 version. We also used the decontam package 1.14.0 version to identify and remove contaminant sequences based on DNA concentration. Specifically, we used the contamdf.prev method, which identifies contaminants based on their prevalence in the negative control sample. This step is crucial to minimizing the impact of contaminants on our results [37]. We then began taxonomic filtering, estimating the prevalence of each taxa in the samples, and agglomeration (phyloseq package).

Statistical manipulations were performed in the R environment. Phyloseq 1.38.0 version was used to calculate measures of microbiome diversity. The Shannon index was used to assess alpha diversity and the Bray–Curtis dissimilarity metric for beta diversity. Differences in alpha diversity were assessed using the Wilcoxon rank sum test. The analysis of variance using the distance matrices (ADONIS) function was used to estimate diversity differences between groups. Analysis of species differences in abundance was performed using the MaAsLin2 package 1.8.0 version, which uses a multivariate regression model [38]. MaAsLin2 includes built-in methods to ensure the robustness of our results, such as false discovery rate (FDR) correction and permutation testing. We used the Benjamini–Hochberg procedure within MaAsLin2 to control for false discovery rate (FDR). This method adjusts *p*-values to account for multiple comparisons, thereby reducing the likelihood of false positives while maintaining statistical power [39]. Permutation testing in MaAsLin2 involves repeatedly shuffling the data and recalculating the test statistics to generate a null distribution that provides empirical *p*-values that help control for multiple comparisons [40].

An adjusted *p*-value of ≤0.05 was considered to be a statistically significant result. Results are presented as mean values ± the standard deviation (SD).

## 3. Results

Analyses of the microbiomes of healthy patients and those diagnosed with IBS-C and IBS-M identified seven types, 10 classes, 24 orders, 39 families, 62 genera, and 101 species of bacteria (Table S1).

### 3.1. Gut Microbiome Composition of the Studied Groups, Defined by Phylum

**Figure 1:**
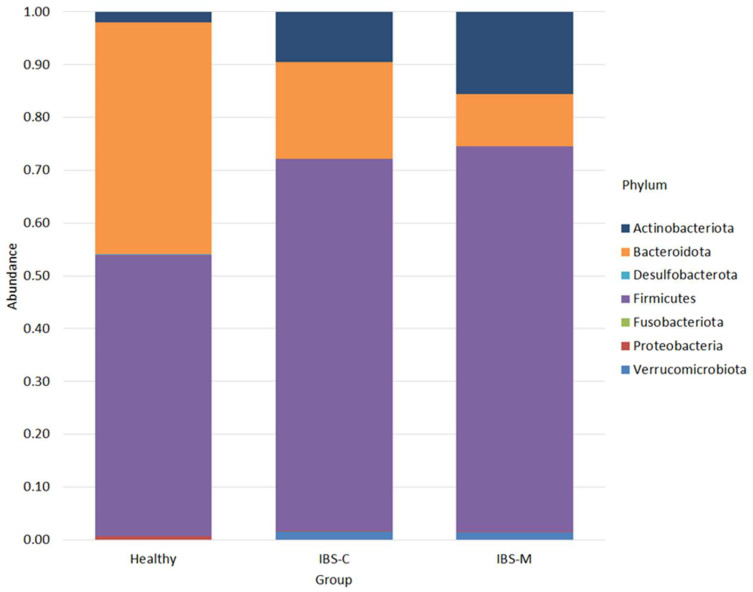
Bacterial phylum found in patients.

The phylum of bacteria detected in the control and study groups are shown in Figure 1.

Firmicutes was the most abundant phylum in all groups, with an abundance of 0.53 ± 0.24 in the healthy group, 0.70 ± 0.14 in the IBS-C group, and 0.73 ± 0.18 in the IBS-M group. Bacteroidota was the next most abundant phylum in both the healthy (0.44 ± 0.25) and IBS-C groups (0.18 ± 0.12), but not in the IBS-M group (0.10 ± 0.10). In patients with IBS-M (0.16 ± 0.19), the second most abundant phylum was Actinobacteriota, which was the third most abundant phylum in both the healthy (0.02 ± 0.01) and IBS-C (0.10 ± 0.09) groups. The next most abundant phylum in both the IBS-C (0.02 ± 0.05) and IBS-M (0.01 ± 0.03) groups was Verrucomicrobiota, whose abundance in the healthy group was 0.0005 ± 0.0006. The abundance of Proteobacteria was 0.006 ± 0.008 in the healthy group and 0.001 ± 0.001 in both IBS subtypes. The Desulfobacterota was present only in the healthy group (0.0002 ± 0.0002).

Differential analysis of abundance showed the presence of statistically significant differences between the healthy group and the IBS-C group, characterized by an increase in the abundance of Firmicutes, Actinobacteriota, and Verrucomicrobiota in the IBS-C group, and a decrease in the abundance of Bacteroidota (*p* = 0.1 in all cases). The same results were observed for the IBS-M group.

### 3.2. Microbiome Composition of the Studied Groups, Defined by Species

**Figure 2:**
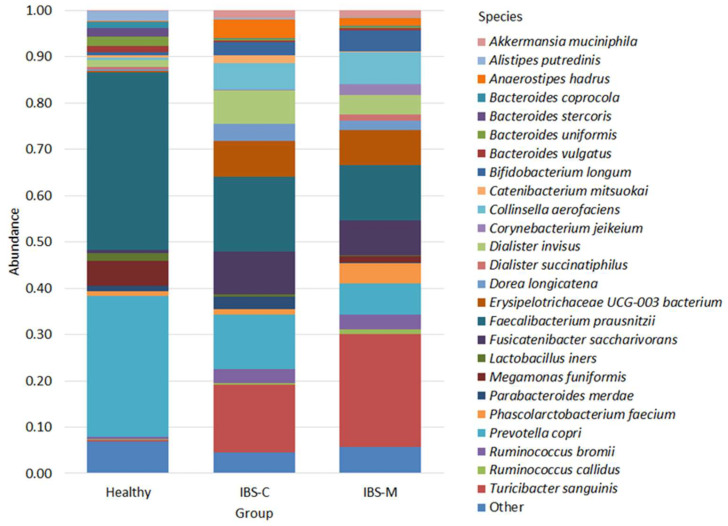
The most abundant species of bacteria found in patients.

The 25 bacterial species were the most abundant members of the microbiome, and their abundance was greater than 0.01 for each study group; all the remaining bacteria were grouped as “Other” (Figure 2).

*Faecalibacterium prausnitzii* was the most abundant species in both the healthy group (0.38 ± 0.12) and the IBS-C group (0.16 ± 0.10), while it was the second most abundant species in the IBS-M group (0.12 ± 0.09). In IBS-M (0.24 ± 0.21), *Turicibacter sanguinis* dominated in abundance, being the next most abundant species in the IBS-C group (0.14 ± 0.18) and poorly represented in the healthy group (0.004 ± 0.005).

In the healthy patients (0.30 ± 0.28), the next most abundant bacterium was *Prevotella copri*, which occupied the same position in the IBS-C group (0.11 ± 0.08); in the IBS-M group, its abundance was 0.07 ± 0.10. The bacteria in the healthy group were also distributed as follows: *Megamonas funiformis* (0.05 ± 0.12), *Alistipes putredinis* (0.02 ± 0.02), *Bacteroides uniformis* (0.02 ± 0.02), *Bacteroides stercoris* (0.02 ± 0.05), *Lactobacillus iners* (0.02 ± 0.01), *Dialister invisus* (0.01 ± 0.02), *Bacteroides vulgatus* (0.01 ± 0.01), *Bacteroides coprocola* (0.01 ± 0.02), *Parabacteroides merdae* (0.01 ± 0.01); the number of the remaining species was less than 0.01.

In the IBS-C group, the distribution was as follows: *Fusicatenibacter saccharivorans* (0.09 ± 0.06), *Erysipelotrichaceae UCG-003* bacterium (0.07 ± 0.11), *Dialister invisus* (0.07 ± 0.10), *Collinsella aerofaciens* (0.06 ± 0.06), *Anaerostipes hadrus* (0.04 ± 0.04), *Dorea longicatena* (0.04 ± 0.03), *Ruminococcus bromii* (0.03 ± 0.04), *Bifidobacterium longum* (0.03 ± 0.05), *Parabacteroides merdae* (0.03 ± 0.08), *Catenibacterium mitsuokai* (0.02 ± 0.03), *Akkermansia muciniphila* (0.02 ± 0.04), *Phascolarctobacterium faecium* (0.01 ± 0.007); the number of the remaining species was less than 0.01.

In the IBS-M group, the distribution was as follows: *Erysipelotrichaceae* UCG-003 bacterium (0.08 ± 0.09), *Fusicatenibacter saccharivorans* (0.07 ± 0.06), *Collinsella aerofaciens* (0.07 ± 0.12), *Bifidobacterium longum* (0.04 ± 0.05), *Phascolarctobacterium faecium* (0.04 ± 0.14), *Dialister invisus* (0.04 ± 0.05), *Ruminococcus bromii* (0.03 ± 0.04), *Corynebacterium jeikeium* (0.02 ± 0.09), *Dorea longicatena* (0.02 ± 0.01), *Anaerostipes hadrus* (0.02 ± 0.01), *Akkermansia muciniphila* (0.01 ± 0.02), *Megamonas funiformis* (0.01 ± 0.04), and *Ruminococcus callidus* (0.01 ± 0.02), the abundance of the rest being less than 0.01.

### 3.3. Alpha and Beta Diversity

**Figure 3:**
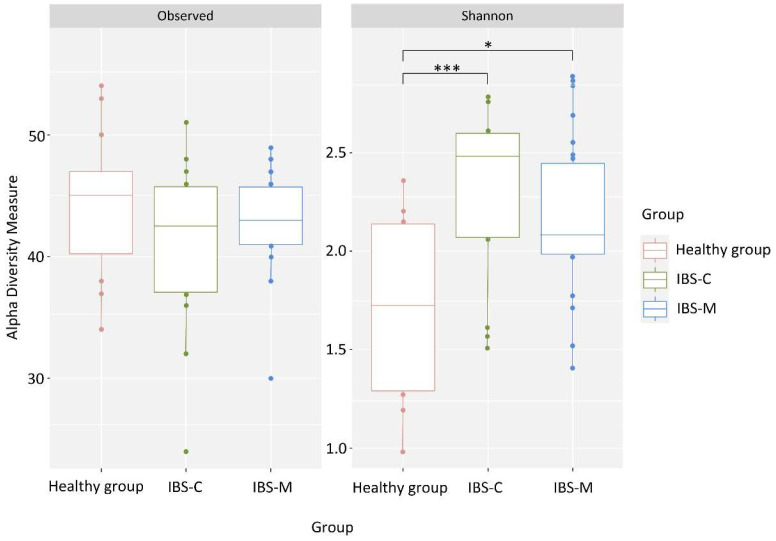
Indicators of alpha diversity of the microbiome of patients with IBS-C and IBS-M and patients without gastrointestinal pathology. * *p* ≤ 0.05, *** *p* ≤ 0.001.

Alpha diversity analysis revealed statistically significant differences between the healthy group and patients with IBS-C (1.78 ± 0.44 vs. 2.28 ± 0.38, *p* = 0.003) and between the healthy group and patients with IBS-M (1.78 ± 0.44 vs. 2.12 ± 0.40, *p* = 0.03) (Figure 3).

**Figure 4:**
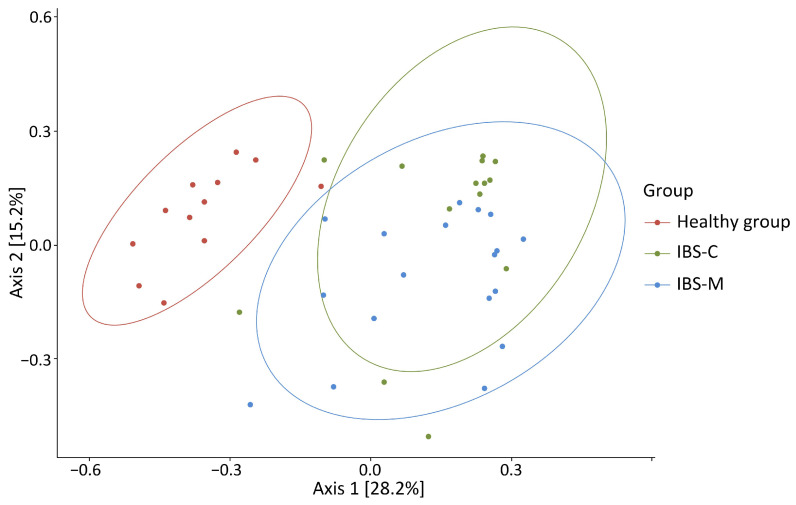
Indicator of beta diversity of the microbiome of patients with IBS-C, IBS-M, and patients without gastrointestinal pathology.

Beta diversity analysis also showed the presence of clustering between the study groups, with the centroid of the microbiome of healthy patients being statistically significantly different from both the IBS-C and IBS-M subgroups, *p* = 0.001 in both cases (Figure 4).

### 3.4. Differential Analysis of Bacterial Species Abundance between Studied Groups

**Figure 5:**
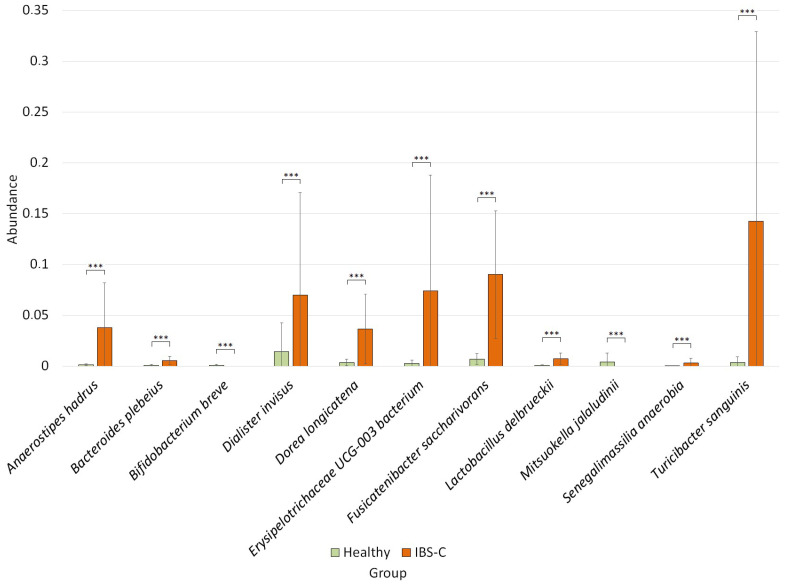
Characteristics of the microbiome in patients with IBS-C, *** *p* ≤ 0.001.

The differential abundance analysis revealed differences at the species level between the healthy group and the IBS-C group (Figure 5).

Thus, in the group of patients with IBS-C, compared to the healthy group, we observed an increase in the number of species *Anaerostipes hadrus* (0.04 ± 0.04 vs. 0.001 ± 0.0009, *p* = 3.20 × 10^−6^), *Bacteroides plebeius* (0.006 ± 0.004 vs. 0.001 ± 0.001, *p* = 2.94 × 10^−5^), *Dialister invisus* (0.07 ± 0.10 vs. 0.01 ± 0.02, *p* = 0.0004), *Dorea longicatena* (0.04 ± 0.03 vs. 0.003 ± 0.003, *p* = 0.0004), *Erysipelotrichaceae UCG-003* (0.07 ± 0.11 vs. 0.003 ± 0.003, *p* = 0.0001), *Fusicatenibacter saccharivorans* (0.09 ± 0.06 vs. 0.01 ± 0.005, *p* = 5.12 × 10^−5^), *Lactobacillus delbrueckii* (0.007 ± 0.005 vs. 0.001 ± 0.0006, *p* = 0.0004), *Senegalimassilia anaerobia* (0.003 ± 0.004 vs. 0.00003 ± 0.0001, *p* = 0.001), *Turicibacter sanguinis* (0.14 ± 0.18 vs. 0.004 ± 0.005, *p* = 6.89 × 10^−6^) and a decrease in the numbers of *Bifidobacterium breve* (0 vs. 0.001 ± 0.0009, *p* = 0.0004) and *Mitsuokella jalaludinii* (0 vs. 0.004 ± 0.008, *p* = 0.001).

**Figure 6:**
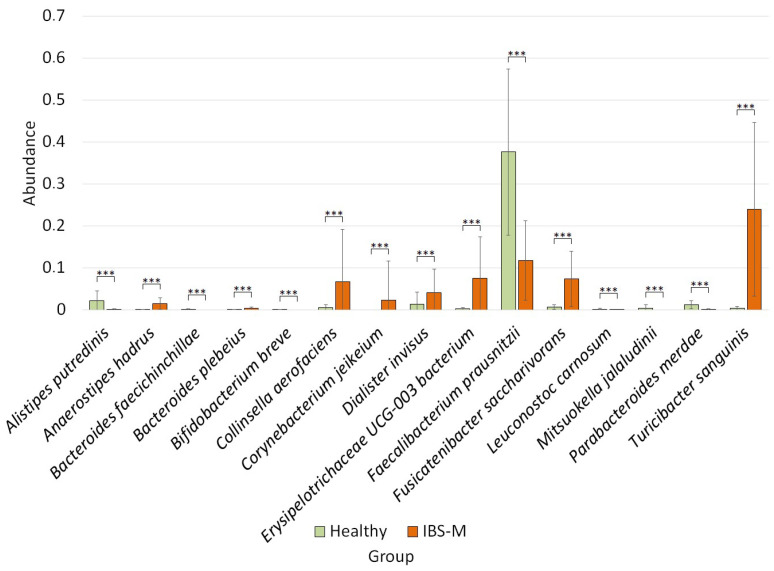
Characteristics of the microbiome in patients with IBS-M, *** *p* ≤ 0.001.

In the group of patients with IBS-M, we observed the following differences (Figure 6).

In the patients with IBS-M, compared to the healthy group, the abundance of *Anaerostipes hadrus* (0.01 ± 0.01 vs. 0.001 ± 0.0009, *p* = 8.08 × 10^−6^), *Bacteroides plebeius* (0.004 ± 0.004 vs. 0.001 ± 0.001, *p* = 0.001), *Collinsella aerofaciens* (0.07 ± 0.012 vs. 0.006 ± 0.006, *p* = 0.001), *Corynebacterium jeikeium* (0.02 ± 0.09 vs. 0, *p* = 0.001), *Dialister invisus* (0.04 ± 0.05 vs. 0.01 ± 0.02, *p* = 0.0005), *Erysipelotrichaceae UCG-003* (0.07 ± 0.09 vs. 0.003 ± 0.003, *p* = 8.08 × 10^−6^), *Fusicatenibacter saccharivorans* (0.07 ± 0.06 vs. 0.007 ± 0.005, *p* = 1.41 × 10^−6^), and *Turicibacter sanguinis* (0.24 ± 0.20 vs. 0.004 ± 0.005, *p* = 6.39 × 10^−8^) was significantly increased. At the same time, the abundance of *Alistipes putredinis* (0.001 ± 0.001 vs. 0.02 ± 0.02, *p* = 4.15 × 10^−5^), *Bacteroides faecichinchillae* (0 vs. 0.001 ± 0.001, *p* = 0.0005), *Bifidobacterium breve* (0 vs. 0.001 ± 0.0009, *p* = 3.59 × 10^−5^), *Faecalibacterium prausnitzii* (0.12 ± 0.09 vs. 0.38 ± 0.019, *p* = 9.17 × 10^−5^), *Leuconostoc carnosum* (0.00004 ± 0.0002 vs. 0.002 ± 0.001, *p* = 8.08 × 10^−6^), *Mitsuokella jalaludinii* (0 vs. 0.004 ± 0.008, *p* = 8.69 × 10^−5^), and *Parabacteroides merdae* (0.001 ± 0.001 vs. 0.01 ± 0.01, *p* = 1.85 × 10^−5^) was decreased.

## 4. Discussion

During this study, 44 fecal samples were sequenced on the Ion Torrent PGM, including 12 from the healthy group of patients and 32 from patients with different types of IBS. Bacteria were identified by species level for each patient. A comparative analysis of the microbiome was also performed to identify bacterial markers characteristic of IBS subtypes, namely IBS-C (14 samples) and IBS-M (18 samples).

There was a difference in alpha diversity scores between patients with IBS-C and IBS-M compared to the healthy group. In general, the microbiome can be characterized as not being very diverse in all groups. There is no consensus on the nature of the change in diversity, with studies showing either opposite results or no significant differences [41]. In our case, the Shannon index was significantly higher in the IBS groups than in the control group, while the total number of OTUs was not significantly different between the groups.

### 4.1. Changes in the Relative Abundance of Bacteria in the IBS-C, IBS-M, and Healthy Groups at the Phylum Level

At the phylum level, we observed the same changes compared to the healthy group in both the IBS-C and IBS-M groups.

We observed an increase in the abundance of Firmicutes in both IBS groups, with a parallel decrease in the abundance of Bacteroidetes, as well as an increase in the Firmicutes/Bacteroidetes (F/B) ratio. Despite the inconsistency of some studies, most studies also observed similar changes in microbiome composition in IBS [30,41,42]. The Firmicutes phylum is dominant in the human gut microbiota and is associated with energy production. It has also been shown to have a potential association with obesity and diabetes [43]. In contrast, the Bacteroidetes phylum produces butyrate, which is associated with indicators of healthy microbiota, as it reduces inflammation and plays a role in normal gut development and repair [43]. In addition, the F/B ratio is thought to be important in signaling the health of the human gut microbiota [44]. Changes in the F/B ratio are associated with diet and obesity [45]. The study by Rajilić-Stojanović et al. (2011) also found a twofold higher F/B ratio in patients with IBS [15]. It is suggested that the high F/B ratio found in some patients with IBS may correlate with the depression and anxiety that often accompany this gastrointestinal disorder [30]. However, despite these findings, there is currently no clear consensus on how these changes relate to IBS. It can be speculated that changes in bacterial types in IBS patients may be associated with changes in epithelial permeability and low-grade inflammation, which are considered possible components of the pathogenesis of IBS [46]. It is worth noting that some studies have found an increase in Bacteroidetes and a decrease in Firmicutes in patients with IBS [47,48]. The discrepancy in results may be due to differences in dietary habits, geographical environment, or even methodological heterogeneity.

We also observed an increase in the abundance of Actinobacteria in patients with both types of IBS compared to controls. The Actinobacteria phylum, one of the major types of intestinal microbiota, plays an important role in maintaining intestinal homeostasis [49]. Changes in the abundance of Actinobacteria have been associated with several diseases, including inflammatory bowel disease [50], ankylosing spondylitis [51], and type 2 diabetes [52]. One study also showed that the type of Actinobacteria was positively correlated with the risk of IBS [53]. However, there was no consistent change in the Actinobacteria phylum across studies, as some studies showed increased [54,55] and others decreased [56] levels in patients with IBS. This certainly points to the important role of this bacterium in the development of different types of IBS, but the specific mechanisms remain to be clarified.

The phylum Verrucomicrobiota was practically absent in healthy patients but was more common in patients with both types of IBS studied. This type of gram-negative bacteria is sometimes observed in the human gut [57]. Some studies suggest that broad-spectrum antibiotic therapy may cause a high degree of colonization of the human gut by Verrucomicrobiota [58]. Some studies, such as ours, have also observed an increase in the abundance of Verrucomicrobiota in the intestines of patients with IBS compared to the healthy group [59,60]. Of particular note is the bacterium *Akkermansia muciniphila*, which was present in trace amounts in the healthy group of patients and was present in both IBS groups at around 1.5%. *A. muciniphila* is thought to have beneficial effects on gut health and metabolic conditions. Many studies highlight the potential of *A. muciniphila* as a therapeutic agent for improving gut health and treating various gastrointestinal disorders [61,62,63]. However, there is also emerging evidence that its abundance may have complex and sometimes adverse effects on gut health. In experimental models, such as mice infected with *Salmonella typhimurium*, the presence of *A. muciniphila* has been found to exacerbate intestinal inflammation. This suggests that although *A. muciniphila* may promote gut health by maintaining mucus integrity and inhibiting pathogenic bacteria, its role may be negatively altered under certain pathological conditions, leading to increased colonic inflammation [64]. In some cases, its interaction with the mucosal immune system may contribute to the development of disease states, especially when the delicate balance of the gut microbiota is disturbed [65]. These findings suggest that although *A. muciniphila* has a beneficial role, its effects may depend on the overall condition of the gut microbiota.

### 4.2. Comparison of Relative Bacterial Abundance between IBS-C, IBS-M, and Healthy Groups at the Species Level

Significant changes were also observed at the species level between the composition of the microbiota of patients with IBS-C and those with IBS-M, compared to healthy patients.

In our study, *Anaerostipes hadrus* was increased in both the IBS-C and IBS-M groups. This bacterium is notable as the first butyrate-producing species that has also been shown to have negative effects on host health [66]. *A. hadrus* is a typical commensal bacterium with a relative abundance of 2–7% in the human gut [67]. Previous studies have confirmed that *A. hadrus* can produce high levels of butyrate from sugars or acetate and lactate metabolized by other bacteria [68]. In addition, *A. hadrus* can also metabolize fructooligosaccharides to support the growth of other bacteria [69]. Because of this, some scientists consider *A. hadrus* to be a beneficial bacterium [70]. However, a recent multi-omics study showed that fatty acid biosynthesis mediated by *A. hadrus* affects the availability of long-chain free fatty acids in the portal circulation and increases liver fibrosis [71]. Another study found a high prevalence of *A. hadrus* in patients with inflammatory bowel disease, IBS, and in patients with colorectal cancer [72,73,74]. The above data suggest that the exact role of *A. hadrus* in maintaining human health remains unclear and requires further research.

The abundance of *Bacteroides plebeius* was also increased in both IBS-C and IBS-M patients. Several literature data have shown an association between *B. plebeius* and the development of IBS, with this bacterium being increased in patients with this disease compared to controls [75]. This species was also common in a group of patients with IBS without constipation [76]. Thus, increased *B. plebeius* may contribute to the gut microbiota dysbiosis, often observed in IBS patients. Dysbiosis can lead to an imbalance in microbial metabolites and affect gut function [15]. An overabundance of *B. plebeius* could also disrupt the intestinal barrier, leading to increased intestinal permeability, a condition often seen in IBS patients [77]. In addition, an increase in this species can lead to excessive gas production, contributing to the bloating and discomfort that are common symptoms of IBS [78]. However, another study showed a decrease in the abundance of the *B. plebeius* species in patients with IBS [79]. In addition, the enrichment of *B. plebeius* species in the gut microbiota was found in the healthy group [80]. The results of our study support an association between the enrichment of *B. plebeius* and the two subtypes of IBS.

The following bacterium is also increased in both groups with different IBS subtypes. *Dialister invisus* is a bacterium capable of producing acetate and propionate [81]. Jossens et al. (2011) reported a reduction in *D. invisus* counts in patients with Crohn’s disease, compared with healthy controls [82]. However, several studies have reported an increase in *Dialister*-like OTUs in adult patients with IBS [30,83]. Increased levels of this bacterium in the oral cavity have also been associated with the severity of IBS [84].

The abundance of *Fusicatenibacter saccharivorans* was increased in both IBS subtypes. There needs to be more data on the role of this bacterium in the human gut and its association with gastrointestinal disease. However, in one study, *F. saccharivorans* was enriched in the microbiome of dysbiotic IBS [85] and an animal model of IBS-D [86]. However, in Crohn’s disease, there is a decrease in the abundance of this species [87].

*Turicibacter sanguinis* is the most studied species of the genus *Turicibacter*, which predominates in the human gut microbiota [88]. *T. sanguinis* may be important for the host’s lipid and steroid metabolism [89]. Previously, this species has been found in animal models with appendicitis and ulcerative colitis [90]. Our study is the first to report a significant increase in the abundance of *T. sanguinis* in patients with IBS-C and IBS-M and its possible role in the pathogenesis of this disease.

At the same time, *Bifidobacterium breve* and *Mitsuokella jalaludinii* species were absent in patients with both IBS subtypes. Members of the genus *Bifidobacterium* are among the first microbes to colonize the human gastrointestinal tract and have a beneficial effect on host health [91]. However, there is a lack of knowledge about the molecular mechanisms that explain these probiotic properties of *Bifidobacterium* [92]. Due to their purported health-promoting properties, *Bifidobacterium* are used as active ingredients in many functional foods. Among these, *B. breve*, originally isolated from infant feces, is one of the most widely used probiotics, improving several gastrointestinal disorders, including IBS, by promoting immunomodulation and protecting the integrity of the intestinal epithelium [93,94]. *Mitsuokella jalaludinii* produces lactic and acetic acid [95], which helps to lower pH, digests amino acids, reduces fumarate to succinate, which can be reduced to propionate, and synthesizes phytase [96,97]. *M. jalaludinii* is known for its ability to ferment carbohydrates to produce short-chain fatty acid (SCFAs), which are essential for gut health. Changes in SCFA levels can affect intestinal motility and inflammation, which are key factors in IBS. Our study is the first to demonstrate the absence of *M. jalaludinii* in patients with IBS.

We also found an increase in bacteria of the genus *Erysipelotrichaceae UCG-003* in patients with both IBS subtypes compared to the control group. Members of the *Erysipelotrichaceae* family have been associated with inflammatory processes in the gut. Dysregulated immune responses and low-grade inflammation are known factors in the pathogenesis of IBS [30].

### 4.3. Specific Changes in the Composition of the Microbiome of the IBS-C Group

We also observed changes in the microbiome that were specific to the IBS subtype studied. For example, in addition to the above changes, we observed an increase in the abundance of *Dorea longicatena*, *Lactobacillus delbrueckii*, and *Senegalimassilia anaerobia* only in the IBS-C group.

*Dorea longicatena* is a species that utilizes carbohydrates and produces gas in the human body, often associated with symptoms such as bloating and abdominal pain [98]. This species is also known to be associated with increased intestinal permeability [99]. Many studies have associated *D. longicatena* with IBS, particularly IBS-D [26,27]. Our study showed that this species is also involved in the pathophysiology of the IBS-C subtype.

*Lactobacillus delbrueckii*, similar to most lactobacilli, is considered a probiotic and may have beneficial effects on the host. Studies have shown that *L. delbrueckii*, in combination with other probiotic bacteria, can reduce intestinal permeability and prevent intestinal barrier dysfunction [100,101]. Recently, however, there have been more reports of increased levels of lactobacilli in the gut microbiota of people with certain conditions (e.g., *H. pylori* infection and IBS), including this study [28,41]. However, most published results are contradictory, suggesting that lactobacilli may not always be beneficial to the host [102,103]. More research is needed to determine whether lactobacilli contribute directly to disease or whether these bacteria are simply adapted to survive in a pro-inflammatory gut environment.

*Senegalimassilia anaerobia* is a Gram-positive anaerobic coccobacillus [104]. To date, there are very few studies of this bacterium in any gastrointestinal disease, except that its abundance is reduced in fecal samples from overweight children [105]. Our study is the first to show an association of this bacterium with IBS-C.

### 4.4. Changes in the Relative Abundance of Bacteria in the IBS-M Group

Only in the IBS-M group, we observed an increase in the abundance of *Collinsella aerofaciens* and *Corynebacterium jeikeium* and a decrease in the number of *Alistipes putredinis*, *Bacteroides faecichinchillae*, *Faecalibacterium prausnitzii*, *Leuconostoc carnosum*, and *Parabacteroides merdae*, compared to the healthy group.

*Collinsella aerofaciens* is the most common Actinobacterium in the human gastrointestinal tract. It is capable of fermenting a range of carbohydrates of plant and animal origin and producing ethanol, short-chain fatty acids, and lactate in the colon [106]. Studies have also confirmed the association of *C. aerofaciens* with several health conditions, including IBS, especially IBS-D [17,27,99,107,108,109].

*Corynebacterium jeikeium* is a Gram-positive bacterium of the human microbiome, is multidrug-resistant and is one of the most clinically important non-diphtheria *Corynebacteria* in the emergency setting [110]. *C. jeikeium* can cause various forms of infection, particularly in immunocompromised patients. *C. jeikeium infection* has been reported in endocarditis, septicemia, meningitis, pneumonia, soft tissue, and urinary tract infections [111,112]. Our study is the first to demonstrate an association of *C. jeikeium* with IBS-M. *C. jeikeium* is known to be an opportunistic pathogen. Its presence in the gut may indicate dysbiosis, which is a common feature of IBS. *Corynebacterium* species can also trigger inflammatory responses. Chronic low-grade inflammation is common in IBS patients, and the presence of pathogenic bacteria could exacerbate this inflammation [113]. In addition, pathogenic bacteria can disrupt the intestinal mucosal barrier, leading to increased intestinal permeability, also known as ‘leaky gut’. This allows bacteria and toxins to enter the bloodstream, which can trigger immune responses and contribute to symptoms [114].

The abundance of *Alistipes* spp. is associated with aging and certain gastrointestinal diseases, such as inflammatory bowel disease and cirrhosis, as well as non-gastrointestinal diseases, such as chronic fatigue syndrome, depression, anxiety, and autism [115]. It has also been reported that patients with IBS have lower levels of *Alistipes* spp. than healthy individuals [15,116], which is consistent with our findings for *Alistipes putredinis*.

There needs to be more literature data characterizing the association of the bacterial species *Bacteroides faecichinchillae* with the pathogenesis of inflammatory bowel diseases. However, it is known that *B. faecichinchillae* was one of the most abundant bacteria in fecal samples from non-obese people compared with obese people [117]. The protective effect of this bacterium has also been previously reported [118]; the absence of it may contribute to the symptoms and development of IBS-M through mechanisms involving gut microbiota dysbiosis, altered SCFA production, immune dysregulation, increased intestinal permeability, and impaired bile acid metabolism [15,77,119,120]. Together, these factors may influence gut motility and inflammation, leading to the alternating symptoms of constipation and diarrhea characteristic of IBS-M.

*Faecalibacterium prausnitzii* is one of the most abundant bacteria in the healthy human gut, and recent research suggests that this bacterium may be beneficial for gut health. The waste products of this member of the gut microbiome provide energy to intestinal epithelial cells through butyrate production and also have immunomodulatory and anti-inflammatory effects [121]. Reports highlight that the abundance of *F. prausnitzii* negatively correlates with the activity of inflammatory bowel diseases, including IBS and colorectal cancer [122,123,124]. Our study showed a threefold decrease in *F. prausnitzii* abundance in the IBS-M group compared to the healthy group.

*Leuconostoc carnosum* is a lactic acid bacterium commonly found in the microbiota of foods, especially meat products [125]. It has previously been reported that *L. carnosum* is found in children with celiac disease [126]. *L. carnosum* produces lactic acid and other SCFAs through fermentation. SCFAs play a crucial role in maintaining gut health by regulating pH, inhibiting pathogenic bacteria, and serving as an energy source for colonocytes. A decrease in SCFA production may disrupt gut homeostasis and contribute to IBS symptoms [120]. Lactic acid bacteria also have anti-inflammatory properties. They can modulate the host’s immune response and reduce inflammation. A decrease in these beneficial bacteria could lead to increased inflammation and worsen IBS-M symptoms [127]. *L. carnosum* contributes to the maintenance of the intestinal mucosal barrier and may inhibit the growth of pathogenic bacteria through the production of bacteriocins and competitive exclusion [128]. This study is the first to show an inverse association between the abundance of this species and IBS-M.

*Parabacteroides* is an important member of the human gut, according to population analyses of variation in the gut microbiome [129]. The presence of *Parabacteroides* is thought to benefit the host, likely through the regulation of immunity, alleviation of inflammation, utilization of broad-spectrum carbohydrates, ability to secrete SCFA, and resistance to some antibiotics [130]. These factors suggest that *Parabacteroides* may use different strategies to survive in the gut, some of which may be beneficial to humans. Previously, a decrease in the number of some representatives of *Parabacteroides* spp. has been observed in the following diseases: inflammatory bowel disease [131], metabolic syndrome [132], obesity [133], gestational diabetes mellitus [134], and some other pathologies. We have shown for the first time a decrease in the abundance of *Parabacteroides merdae* in IBS-M.

It is also important to note that we did not find statistically significant differences in the microbiome composition between IBS-C and IBS-M.

Regarding the IBS-D subtype, which was not studied in this paper but was the focus of our previous work, an association of IBS with the genera *Streptococcus* and *Haemophilus* was found, which are mainly opportunistic pathogens and, according to literature data, are associated with the development of IBS [135]. In this study, no association was found between IBS-C and IBS-M subtypes and these bacteria. However, based on other data, some bacteria, such as *F. saccharivorans*, *D. longicatena* and *C. aerofaciens* have previously been associated with the IBS-D subtype, and our study showed their association with other IBS subtypes as well.

It is certainly known that differences in the composition of the microbiome between the control group and patients with IBS do exist, but the microbiome specific to IBS has not been clearly identified. Additionally, to our knowledge, no significant differences have been found between the IBS-C and IBS-M subtypes to date. This was one of the focuses of this study, the results of which show the potential clinical relevance of microbiome monitoring in patients with IBS. The data described will provide a basis for future research, as some of the findings were observed for the first time and certainly require further study in larger cohorts of patients.

This study has limitations, the main one being the small size of the study sample. Another limitation of our study is the uneven gender distribution between the study groups. Specifically, the healthy group had only male members, whereas the IBS groups had both male and female participants. This imbalance could influence the microbial composition due to known gender differences in the microbiome. The over- or under-representation of one gender in the groups could affect the generalizability of our findings. In addition, this study lacks the assessment of IBS disease activity, which could perhaps be addressed through the use of specific questionnaires. We have no information on the ‘severity’ of IBS, only on the duration of the disease. An additional limitation is the method used to identify the microbiome. This study lacks data on the non-invasive markers of inflammation and their association with the microbiota. The sequencing platform used and the approach of targeted sequencing of the *16S rRNA* gene have some restrictions, such as the inability to identify microbiome components, including eukaryotes and viruses, and lower sequencing accuracy compared to platforms based on the shotgun method. Another limitation is the lack of comparison with other available microbiome databases. 

## 5. Conclusions

In this study, we analyzed the microbiome composition of feces from patients with two subtypes of IBS, including IBS-C and IBS-M, as well as a healthy group of individuals, to identify the microbiome patterns of this gastrointestinal pathology. This study was conducted using the Ion Torrent PGM next-generation sequencing platform.

Differences in microbiome composition were found between the two IBS subgroups and the healthy group. These differences consisted of increased alpha diversity in the patients with IBS-C and IBS-M, compared to the healthy group. Differences in beta diversity were also noted. The microbiome differences were characterized at both the phylum and species levels. At the phylum level, an increased Firmicutes/Bacteroidetes ratio, an increased abundance of Actinobacteria, and the presence of Verrucomicrobiota were observed in both IBS subtypes. At the species level, microbiome aberrations were identified that were broadly consistent across both IBS-C and IBS-M and in accordance with previous studies. Changes in some bacterial species were characteristic of only one of the IBS subtypes, while there were no statistically significant differences in microbiome composition between IBS-C and IBS-M. This study was also the first to show the association of *Turicibacter sanguinis*, *Mitsuokella jalaludinii*, *Erysipelotrichaceae UCG-003*, *Senegalimassilia anaerobia*, *Corynebacterium jeikeium*, *Bacteroides faecichinchillae*, *Leuconostoc carnosum*, and *Parabacteroides merdae* with IBS.

This study advances our understanding of the contribution of individual members of the microbiome to the development of IBS overall and its subtypes, IBS-C and IBS-M. A deep understanding of the role of individual microorganisms in the development of IBS will bring us closer to early diagnosis of this gastrointestinal pathology, as well as the development of individualized therapy based on correcting microbiome imbalances.

## Figures and Tables

**Table 1:** Characteristics of study groups.

Group	Height ± SD, cm	Weight ± SD, kg	BMI ± SD	Age ± SD	Duration of the Disease
Control	180.92 ± 8.04	77.83 ± 7.64	23.79 ± 2.19	30.41 ± 6.42	—
IBS-C	178.43 ± 8.62	74.14 ± 5.88	23.96 ± 2.61	32.67 ± 6.67	5.36 ± 4.43
IBS-M	170.80 ± 10.27	65.11 ± 12.12	22.59 ± 2.02	30.14 ± 6.79	5.50 ± 3.49

## Data Availability

Raw sequencing data are available in the NCBI BioProject database (BioProjectID: PRJNA 817720).

## Supplementary Materials

The following supporting information can be downloaded at: https://www.mdpi.com/article/10.3390/microorganisms12071414/s1, Table S1: A complete list of the bacterial taxa identified for the samples under investigation.

## Abbreviations

*16S rRNA*	*16S ribosomal RNA*;
ASVs	amplicon sequence variants;
BSFS	Bristol Stool Form Scale;
dsDNA	double-stranded DNA;
FDR	false discovery rate;
IBD	Inflammatory bowel disease;
IBS	Irritable bowel syndrome;
IBS-C	constipation-predominant IBS;
IBS-C	constipation-predominant IBS;
IBS-D	diarrhoea-predominant IBS;
IBS-M	mixed fecal IBS;
NCBI	National Center for Biotechnology Information;
OTU	Operational taxonomic unit;
PCR	Polymerase chain reaction;
PGM	Personal Genome Machine;
SCFA	Short-chain fatty acid;
SD	standard deviation.
